# Retinal blood flow dysregulation precedes neural retinal dysfunction in type 2 diabetic mice

**DOI:** 10.1038/s41598-021-97651-3

**Published:** 2021-09-15

**Authors:** Junya Hanaguri, Harumasa Yokota, Masahisa Watanabe, Satoru Yamagami, Akifumi Kushiyama, Lih Kuo, Taiji Nagaoka

**Affiliations:** 1 Division of Ophthalmology, Department of Visual Sciences, Nihon University School of Medicine, 30-1 Oyaguchi-Kamicho, Itabashi-ku, Tokyo, 173-8610 Japan; 2 Department of Pharmacotherapy, Meiji Pharmaceutical University, Tokyo, Japan; 3 Department of Medical Physiology, Texas A&M University Health Science Center, Bryan, TX USA

**Keywords:** Circulation, Experimental models of disease, Eye diseases, Metabolic disorders

## Abstract

We investigated and compared the susceptibility of retinal blood flow regulation and neural function in mice developing type 2 diabetes. The longitudinal changes in retinal neuronal function and blood flow responses to a 10-min systemic hyperoxia and a 3-min flicker stimulation were evaluated every 2 weeks in diabetic db/db mice and nondiabetic controls (db/m) from age 8 to 20 weeks. The retinal blood flow and neural activity were assessed using laser speckle flowgraphy and electroretinography (ERG), respectively. The db/db mice had significantly higher blood glucose levels and body weight. The resting retinal blood flow was steady and comparable between two groups throughout the study. Hyperoxia elicited a consistent decrease, and flicker light an increase, in retinal blood flow in db/m mice independent of age. However, these flow responses were significantly diminished in db/db mice at 8 weeks old and then the mice became unresponsive to stimulations at 12 weeks. Subsequently, the ERG implicit time for oscillatory potential was significantly increased at 14 weeks of age while the a-wave and b-wave amplitudes and implicit times remained unchanged. The deficiencies of flow regulation and neurovascular coupling in the retina appear to precede neural dysfunction in the mouse with type 2 diabetes.

## Introduction

Recent clinical observations for diabetes revealed that prior to the development of vascular lesions and visible retinopathy, retinal thinning occurs^1,2^. These results support the concept that diabetes (type 1 and type 2) evokes early neurodegeneration in the retina, which appears to occur concurrently or before the structure and morphological changes in retinal vasculature^3,4^. Interestingly, abnormal retinal circulation was also found in patients with type 1^5,6^ and type 2^7^ diabetes before visible retinopathy developed. Thus, early detection and treatment of abnormal retinal circulation may be a valuable strategy to minimize the development of diabetic retinopathy. However, in contrast to vascular lesions, the information on the temporal change of retinal vasomotor activity and blood flow regulation during the progression of diabetes is not currently available, and the relative susceptibility of retinal circulation versus neuroretinal function during diabetic insult remains unknown.

Retinal blood flow is known to be intrinsically regulated to maintain proper retinal function^8,9^. Systemic hyperoxia (100% oxygen inhalation) produces a reduction of retinal blood flow, a response mediated by the released vasoconstrictor endothelin-1 (ET-1)^10^ from glia cells^11^, linking oxygen homeostasis to retinal blood flow regulation. In addition, Riva et al. reported that retinal blood flow is increased when a flickering light stimulates the neuronal retina^12^. This hyperemic response was thought to be mediated by the linkage of neural activity and metabolism to blood flow regulation, i.e., neurovascular coupling, in the retina^13^. Clinical studies reported that diabetes blunts retinal blood flow responses to systemic hyperoxia^14^ and flicker light^15^, corresponding to the extent of pathological neovascularization and increased stage of retinopathy. However, it is unclear whether the altered vascular structure leads to flow dysregulation in diabetes, or vice versa. Interestingly, in both type 1 and type 2 diabetes, a close association between impairment of the vascular response to flicker light and abnormal ERG was noted in patients without retinopathy^16^. Moreover, a subtle decrease in capillary density was found to associate with neural function alterations in patients with type 2 diabetes who did not have visible lesion in the retina^17^. However, the extent of the relationship between these abnormalities remains unknown. It is worth noting that the aforementioned clinical studies were cross-sectional with various ages of diabetes. Thus, there is a need to determine the exact relationship between the alterations of neuronal activity and vascular function during progression of type 2 diabetes.

In the present study, using a longitudinal approach, we examined the time course of development of flow dysregulation versus neural dysfunction in type 2 diabetes in the same subject. This study examined changes in retinal blood flow in response to hyperoxia and flicker light with laser speckle flowgraphy (LSFG) and evaluated neural retina function with ERG using a genetic type 2 diabetes mouse model, db/db^18^, between the ages 8 weeks and 20 weeks old. We tested the hypothesis that retinal blood flow dysregulation precedes the development of retinal neuronal dysfunction in type 2 diabetes.

## Results

### Longitudinal assessment of systemic and ocular parameters

**Figure 1:**
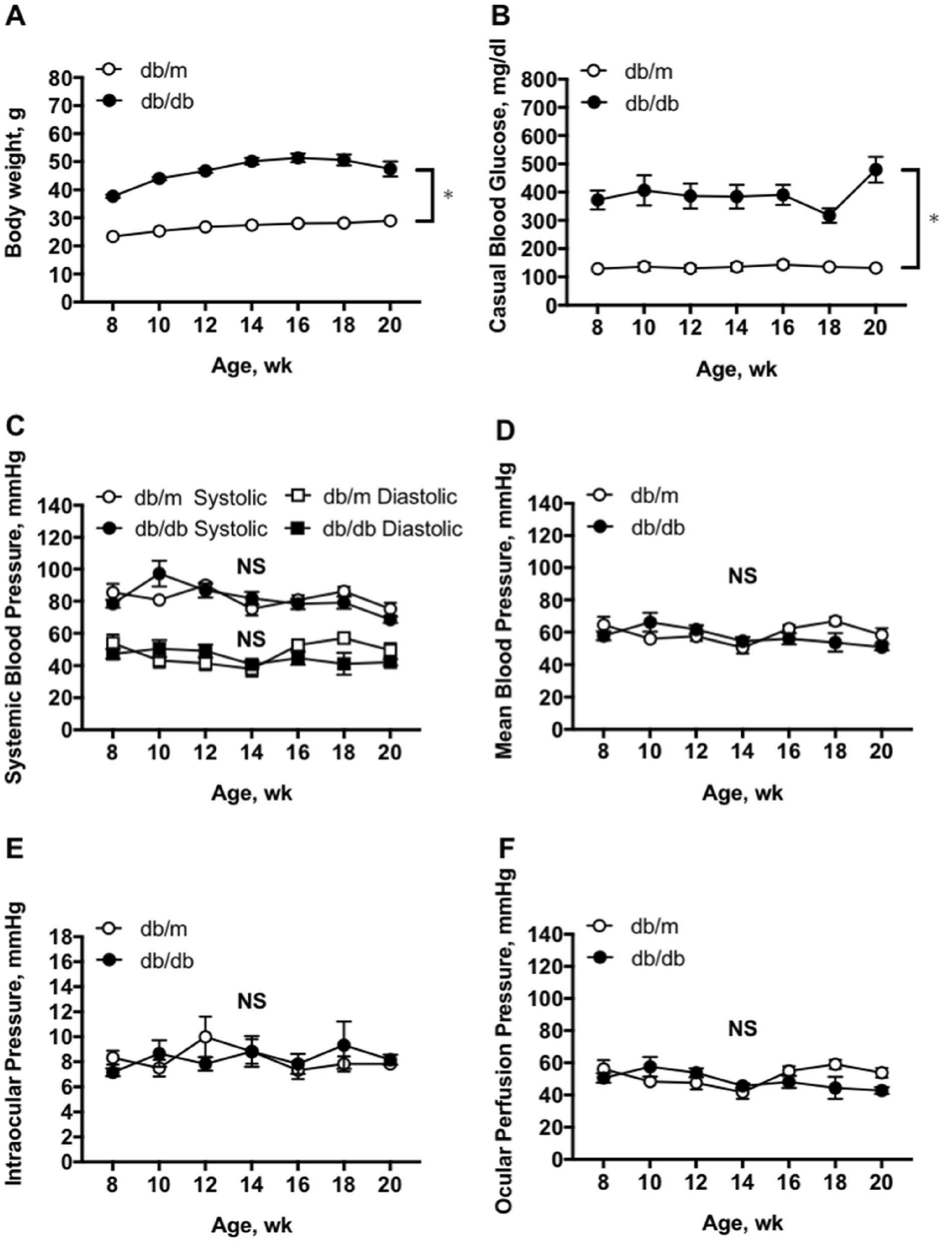
Average systemic and ocular parameters in db/db and db/m mice from 8 to 20 weeks of age. Significant increases were observed in body weight (**A**) and blood glucose (**B**) in db/db mice (n = 6) compared with nondiabetic db/m mice (n = 6) by two-way repeated measures ANOVA. In contrast, no significant differences were observed in systemic blood pressure (**C**), mean arterial blood pressure (**D**), intraocular pressure (**E**), and ocular perfusion pressure (**F**) between two animal groups during follow-up period. Data are expressed as the mean ± SEM; au = arbitrary unit; *P < 0.05 between groups; NS = not significant between groups.

Bodyweight was significantly higher in the db/db mice than the nondiabetic db/m mice during the experiments (two-way repeated measures ANOVA; Fig. 1A). The blood glucose levels were relatively constant in all mice throughout the study, with about three folds higher in resting blood glucose in db/db mice (two-way repeated measures ANOVA; Fig. 1B). The systolic blood pressure (SBP), diastolic blood pressure (DBP), and mean arterial blood pressure (MABP) were not different between db/m and db/db mice (Fig. 1C,D; two-way repeated measures ANOVA). The intraocular pressure (IOP) and ocular perfusion pressure (OPP) were not different between two groups of mice (Fig. 1E,F; two-way repeated measures ANOVA). These systemic and ocular parameters were not affected by age (one-way repeated measures ANOVA).

### Longitudinal assessment of resting retinal blood flow

**Figure 2:**
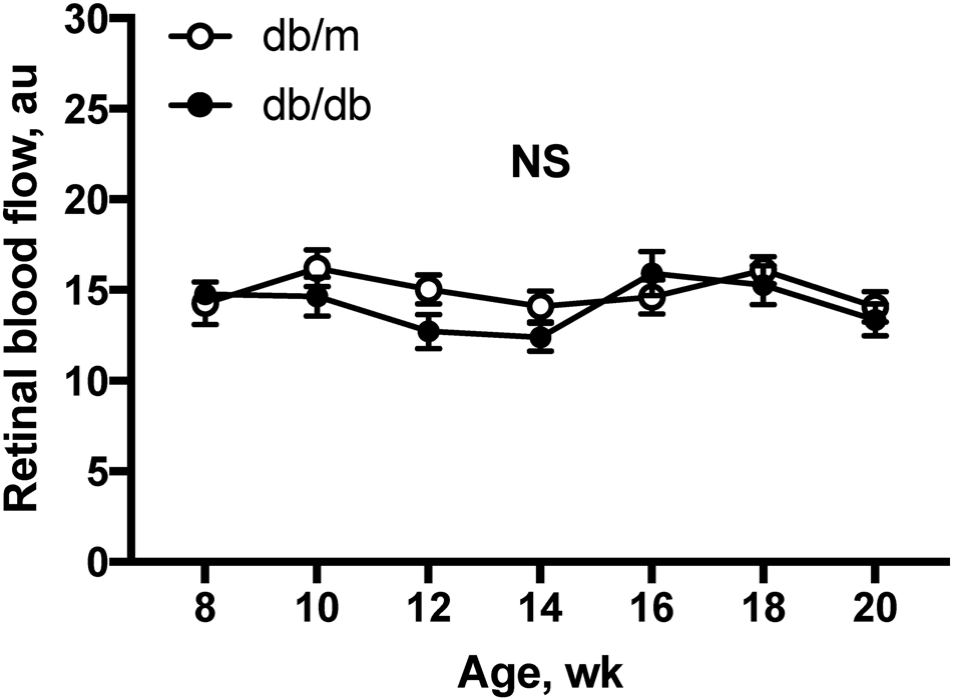
Retinal blood flow in db/db and db/m mice from 8 to 20 weeks old. Retinal blood flow (a.u) remained stable in both groups throughout, by one-way repeated measures ANOVA (P = 0.37 for db/m mice and P = 0.47 for db/db mice). No differences in resting retinal blood flows were observed (two-way repeated measures ANOVA). *NS* not significant between groups and within group.

Figure 2 presents the stability of resting retinal blood flow. Both db/m and db/db mice exhibited a steady resting retinal blood flow throughout the experimental period (8 to 20 weeks) with no difference between the groups (two-way repeated measures ANOVA).

### Longitudinal assessment of retinal blood flow response to systemic hyperoxia

**Figure 3:**
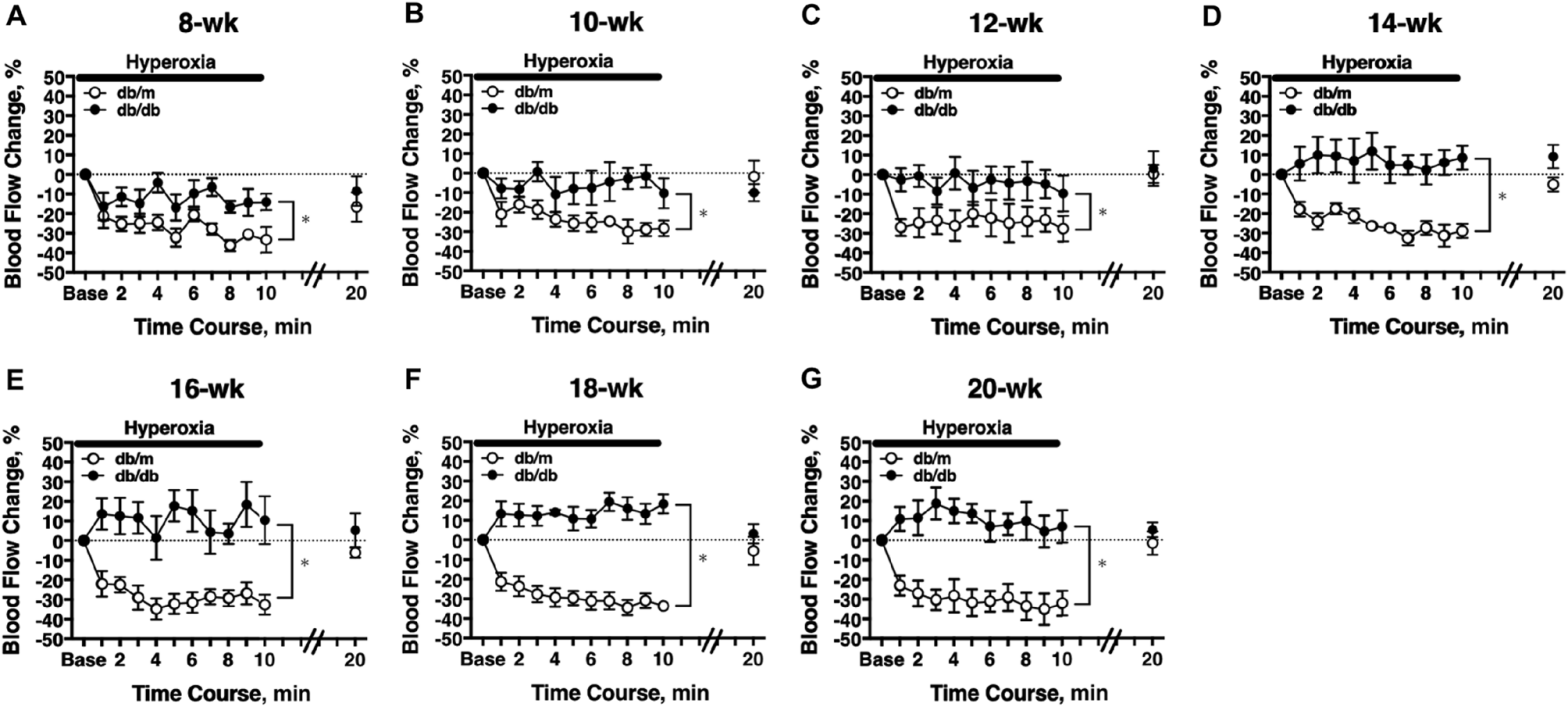
Retinal blood flow response to systemic hyperoxia. Longitudinal assessment of retinal blood flow in db/db and db/m mice from 8 to 20 weeks old. Retinal blood flow was significantly reduced from baseline during hyperoxia in db/m control mice (n = 6) from 8 weeks (**A**) to 20 weeks (**G**) of age. Reduction of retinal blood flow from baseline was observed in 8-week old db/db mice (A). No significant changes in retinal blood flow were noted in db/db mice (n = 6) at 10 weeks (**B**) and older (**C**–**G**) (one-way repeated measures ANOVA). The hyperoxia-induced flow response was significantly blunted in db/db mice for each age studied (two-way repeated-measures ANOVA). Following termination of hyperoxia, return of retinal blood flow to baseline levels occurred within 10 min, and no difference was observed between groups (two-way repeated measures ANOVA). *P < 0.05 between groups; Solid bar = period of hyperoxia.

On experiment day-1, after the measurement of baseline retinal blood flow, a 10-min systemic hyperoxia was imposed. The retinal blood flow decreased significantly with systemic hyperoxia in db/m mice at 8 weeks of age (one-way repeated measures ANOVA; Fig. 3A). This flow response pattern was consistently observed along with the growth of the animal to 20 weeks (Fig. 3B–G). The retinal blood flow returned to resting levels within 10 min after cessation of systemic hyperoxia (Fig. 3A–G). In 8 weeks old db/db mice, a reduction in retinal blood flow in response to hyperoxia was also observed; however, the response was significantly blunted compared to that in db/m mice (two-way repeated measures ANOVA; Fig. 3A). The hyperoxia-induced flow reduction was absent in db/db mice at 10 weeks, 12 weeks, and 14 weeks old (Fig. 3B–D). As the db/db mice grew older, there was a tendency to increase retinal blood flow in response to hyperoxia (Fig. 3E–G). No difference was observed in resting blood flows between db/db and db/m mice 10 min after the hyperoxia cessation.

### Longitudinal assessment of retinal blood flow response to flicker stimulation

**Figure 4:**
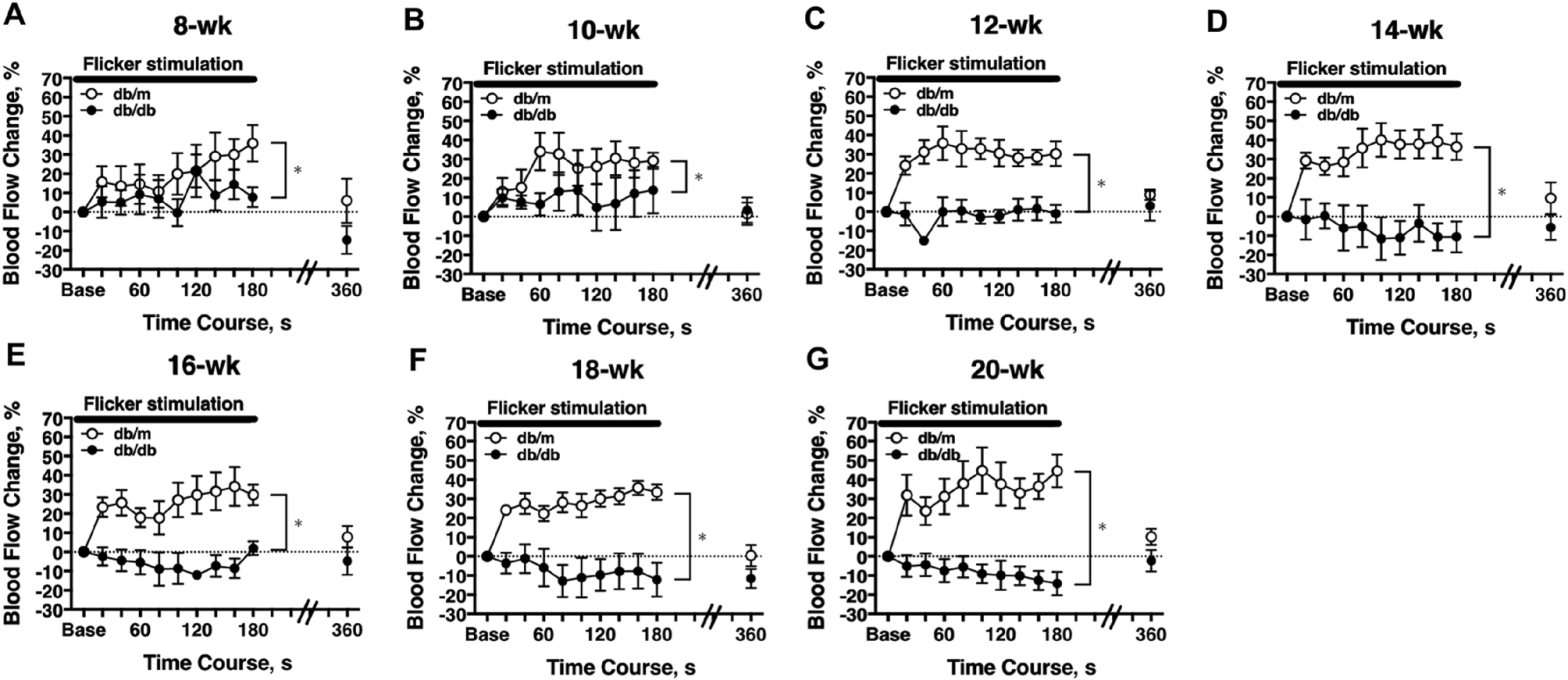
Retinal blood flow response to flicker stimulation. Retinal blood flow was longitudinally assessed in db/db (n = 6) and db/m (n = 6) mice from 8 to 20 weeks of age. During the period of flicker light stimulation, retinal blood flow increased significantly in db/m mice from 8 weeks (**A**) to 20 weeks (**G**) of age and in db/db mice at 8-week (**A**) and 10-week (**B**) old. When the db/db mice grew older (**C**–**G**), retinal blood flow changes in response to flicker light were not significant (one-way repeated measures ANOVA). The flicker-induced flow response was significantly blunted in db/db mice for each age studied (two-way repeated measures ANOVA). The retinal blood flow in db/m mice returned to baseline at three minutes after termination of light stimulation, and there was no difference between groups (two-way repeated -measures ANOVA). *P < 0.05 between groups; Solid bar = period of flicker stimulation.

On experiment day-2, the temporal change of retinal blood flow in response to a 3-min flicker light stimulation was assessed. In 8-week-old db/m mice, the flicker light caused a slow and steady increase in retinal blood flow by 30% above baseline (one-way repeated measures ANOVA; Fig. 4A). When the mice grew older, from around 10 weeks, the flow was increased promptly and then stabilized at about 30% above baseline after 60 s of light stimulation (Figs. 4B–G). The blood flow returned to baseline within 3 min after flicker stimulation cessation (Figs. 4A–G). In db/db mice, the flow response to flicker light was blunted at younger ages, i.e., 8-week (Fig. 4A) and 10-week old (Fig. 4B). By 12 weeks of age, the mice became unresponsive to stimulation (Fig. 4C two-way repeated-measures ANOVA). There was a tendency of reversing the light-stimulated flow response in db/db mice beyond 12 weeks old (Figs. 4D–G). At 3 min after cessation of the flicker light stimulation, no difference was observed in resting blood flows between db/db and db/m mice.

### Longitudinal assessment of ERG parameters

**Figure 5:**
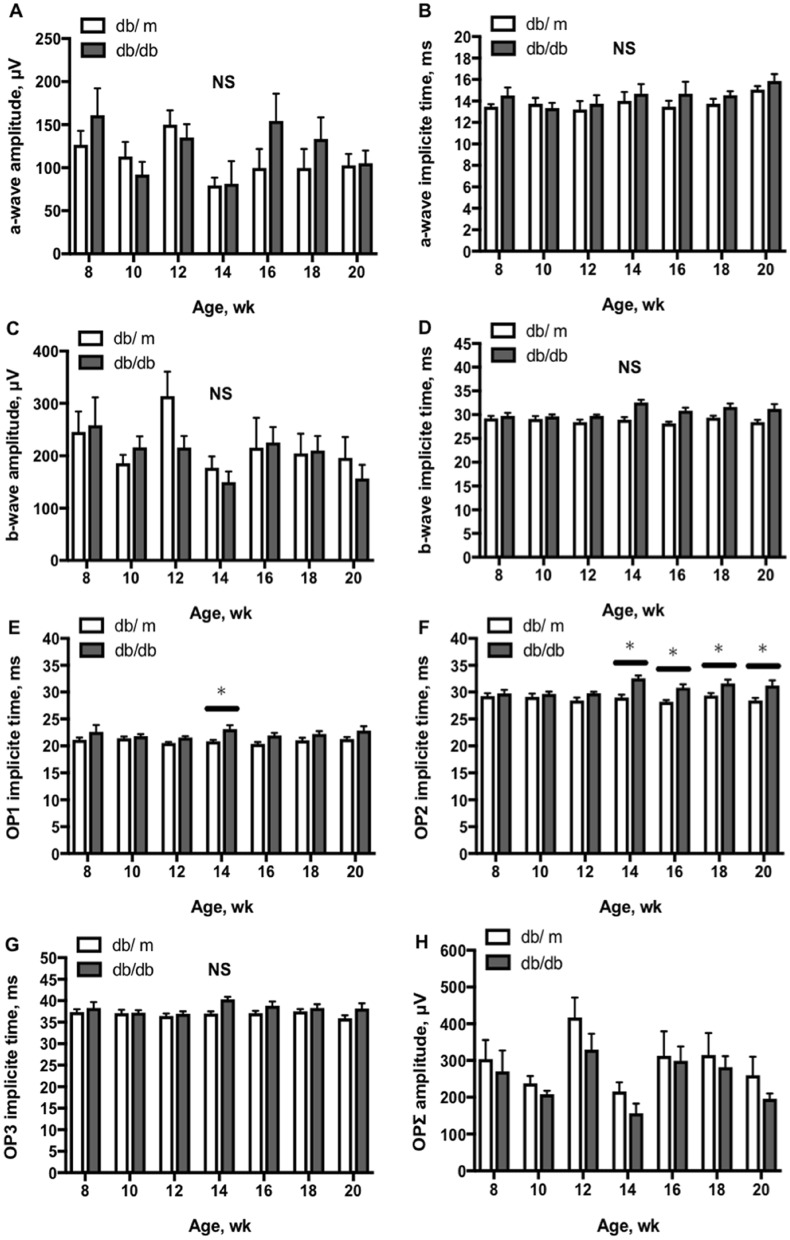
Longitudinal assessment of ERG in db/db and db/m mice from 8 to 20 weeks of age. There were no significant differences in implicit time and amplitude of a-wave (**A**,**B**) and b-wave (**C**,**D**) between db/m (n = 6) and db/db (n = 6) mice. There were no significant changes in the implicit times of OP3 (**G**) waves and the total amplitudes of OP-waves (ΣOP) (H) between db/m and db/db mice (two-way repeated -measures ANOVA). The implicit times of OP1 (**E**) were significantly increased in db/db mice at 14 weeks old and the implicit times of OP2 waves (**F**) were significantly increased in db/db mice from 14 to 20 weeks old (two-way repeated -measures ANOVA). *P < 0.05, between groups.

On experiment day-3, the ERG was assessed. There were no significant changes in amplitude and implicit time of a-wave and b-wave ERG in both db/m and db/db mice with age (Fig. 5A–D; one-way and two-way repeated-measures ANOVA). While no significant differences were found between db/m and db/db mice in the implicit time of OP3 (Fig. 5G) and the ΣOP amplitude (Fig. 5H), the implicit times of OP1 were significantly increased in db/db mice at 14 weeks old (Fig. 5E; two-way repeated-measures ANOVA). From 14 to 20 weeks, post hoc comparison with Holm-Sidak test showed that the implicit time of OP2 was significantly prolonged in db/db mice compared with db/m mice (Fig. 5F).

## Discussion

Our present findings provide the first longitudinal data on the deterioration of retinal blood flow regulation before the development of neural deficiency during type 2 diabetes progression in mice. We found that the resting retinal blood flow was not altered in db/db mice from 8 to 20 weeks of age. However, the retinal blood flow responses to systemic hyperoxia and flicker light stimulation were compromised in the early stage of diabetes before the presence of ERG abnormality.

A reduction of resting retinal blood flow has been reported, without a noticeable change in retinal arterial and venous diameters, in rats after one week of streptozotocin-induced diabetes^19,20^. However, no changes in resting retinal blood flow and vascular diameters were found in Akita type-1 diabetic mice from 5 to 13 weeks of age^21^. It is unclear whether differences in species, age, and/or the type 1 diabetes model contributed to the inconsistent results. Using magnetic resonance imaging technology, retinal blood flow was also found unaltered in Akita mice at 10 weeks old, but a significant reduction of resting flow associated with visual deficiency was noted at older ages (i.e., 30 weeks of age)^22^. Prolonged diabetes appears to contribute to reducing resting retinal blood flow. However, the temporal relationship between the flow alteration and the observed neural deficiency remains unclear as only two time points (i.e., 10 and 30 weeks) were examined in the above study. We measured the global change of retinal blood flow in type 2 diabetes every two weeks in the same subjects. Our results showed a steady resting retinal blood flow from 8 to 20 weeks of age with no differences between db/db mice and their age-matched controls. Although our current study did not extend the flow assessment beyond 20-week-olds, our findings indicate that type 2 diabetes, up to early adulthood, has little impact on the resting retinal blood flow.

The neurovascular coupling mechanism that optimally regulates retinal blood flow to match oxygen demand and metabolic activity of the retinal tissue is well-established^8,23^. The retinas respond to systemic hyperoxia with reduction of retinal perfusion^10,11,24,25^ through the release of a potent vasoconstrictor ET-1^10,11^ from neural glia cells^11^ and the subsequent activation of ET-1 type A receptors (ET_A_R) in retinal blood vessels^10,26,27^. Administration of ET-1 significantly reduces retinal blood flow in healthy humans without affecting retinal arterial and venous diameters, suggesting the main action of ET-1 in the retinal microcirculation^28^. ET-1 does not contribute to the maintenance of retinal vascular tone or diameter in healthy human subjects during rest^28^. However, the ET-1 levels in vitreous fluid^29–31^, retinal tissue^32,33^, and plasma^34–36^ are elevated in subjects with diabetes. Interestingly, a sevenfold increase in vascular ET-1 mRNA was reported in type 1 diabetic mice^37^. Administration of ET_A_R blockers in animals with early diabetes prevents a decrease in the retinal blood flow, suggesting the contribution of upregulated ET-1 system to early vascular complication in diabetic retinas^38,39^.

In the present study, we did not observe a decrease in resting retinal blood flow; however, the blood flow response to hyperoxia was significantly blunted in diabetic mice. Initially, the extent of flow reduction was reduced around 8 weeks old (early stage of diabetes). Following this, the retinal circulation became unresponsive to stimulation (10–12 weeks) and tended to reverse the flow response (i.e., increase in flow) at the later stages of diabetes (14–20 weeks) (Fig. 3). Because ET-1 has been shown to be responsible for the reduction of retinal blood flow during hyperoxia^10,40^, the observed impairment of this flow response in the present study might be explained by the diminished vascular responsiveness to ET-1, possibly due to ET_A_R desensitization^34^ in vascular smooth muscle cells^41–46^ and/or pericytes^47^ which are pre-exposed to the elevated level of ET-1 in diabetes. In line with this speculation, a reduced mesenteric^35^ and retinal^48^ vascular responsiveness to ET-1 has been reported in diabetic rats. Moreover, the hyperglycemic insult might also contribute to the reduction of flow response to hyperoxia by impairing ET-1-mediated biochemical signaling in retinal pericytes^49^. Collectively, these findings suggest that the diabetic insult may gradually compromise the coupling between glial cells and the vasoconstriction to ET-1 at downstream terminal microvessels, where the pericyte is abundant and dominant for blood flow regulation^50^. Our findings are in line with observations in other studies of reduced retinal blood flow response to hyperoxia in patients with diabetes, with or without retinopathy^24,51,52^. Further mechanistic studies are warranted to investigate how hyperglycemia and diabetes exert negative impacts on the retinal microvascular function related to ET-1 overproduction.

Riva et al. were the first to show that the increase of optic nerve head blood flow evoked by a flickering light is associated with the reduction of retinal pO_2_, possibly due to increased metabolic activity of ganglion cells^12^. The increased neuronal activity can consequently increase retinal blood flow by triggering release of vasodilators such as nitric oxide (NO) and/or arachidonic acid metabolites^53,54^. Clinical studies found that the vasodilation elicited by flicker light is reduced in patients with diabetes, correlating with an advanced stage of retinopathy^15,55^. In fact, some patients with diabetes (both type 1 and type 2) showed reduced flicker-induced flow response before the clinical appearance of retinopathy^15^, possibly reflecting neural dysfunction in the inner retina^16^. However, the development of flow dysregulation in response to flicker light, in relation to neural dysfunction, during the progression of diabetes is not known. There also has been no report concerning the impact of type 2 diabetes on retinal blood flow regulation in db/db mice, an animal model widely used for retinal disease research. We found that the hyperemia induced by flicker light was gradually diminished with age in db/db mice (Fig. 4). Interestingly, the compromised flow response was already present in the 8-week-old mice, the youngest age studied, where blood glucose level was already significantly elevated (Fig. 1). Because the onset of hyperglycemia in db/db mice has been reported to be at 4 weeks of age^18^, it is likely that the deterioration of flow regulation might have begun at early diabetes around 4–6 weeks old. This speculation is supported by finding vasomotor dysregulation of both retinal arterioles^56,57^ and venules^29^ after induction of type 1 diabetes for only 2 weeks. Although the NO and arachidonic acid metabolites can participate in flicker-induced vasodilation^53^ and increased retinal blood flow^54^, the mechanisms underlying neurovascular uncoupling and flow dysregulation in the diabetic retina are incompletely understood. Interestingly, a recent study in diabetic pigs demonstrated the impairment of endothelium-dependent NO-mediated dilation of retinal arterioles by upregulated vascular arginase^57^, an enzyme that competes with NO synthase for their common substrate L-arginine, and thus consequently compromises NO production and vasodilation^58^. Because the vasodilation mediated by arachidonic acid^59^ appears to be intact in retinal arterioles isolated from diabetic animals^57^, the observed retinal blood flow dysregulation in response to flicker light is likely due, in part, to NO deficiency. Nonetheless, contribution from other factors cannot be excluded because a progressive reduction of neuroretinal thickness and the loss of ganglion cells become apparent in db/db mice at 16- and 24-week of age^18^. These structural changes may worsen neurovascular uncoupling and completely exhaust retinal blood flow regulation in advanced stages of diabetes (i.e., age 10–12 weeks and older) as shown in the present study (Figs. 4 and 5).

It has been reported that OPs are the most sensitive ERG parameters, reflecting the changes in microvascular function^9,60^. The OPs are likely to originate from the inner retinal neuron activity, which is known to be vulnerable to ischemia^60^. The OP electroactivity could also be derived from retinal glial cells, amacrine, and interplexiform cells, which are sensitive to hyperglycemic insults^61^. Our results agree with previous clinical studies showing that the prolonged OP implicit times are present earlier than the decrease of amplitudes in diabetic patients^60^. We further showed that the onset of OP abnormality (at 14-week old; Fig. 5) occurred at the time after the complete exhaustion of retinal blood flow regulation (at 10- to 12-week old; Figs. 3 and 4), suggesting a possible link of flow dysregulation to the development of neural dysfunction in the inner retina during diabetic insult. On the other hand, we found that there were no significant alterations in both a- and b-wave amplitudes and implicit times in db/db mice (Fig. 5). These results are inconsistent with a previous report by Bogdanov et al. on the appearance of reduced b-wave amplitudes and prolonged implicit times in db/db mice at 16 and 24 weeks of age^18^. Although the reason for this discrepancy is unclear, we noticed that the variability of b-wave amplitudes was greater than the variability of its implicit times across different ages and the coefficient of variation of b-wave amplitudes in our study was higher than that of Bogdanov’s study at 16 and 24 wks. The difference in the number of animals studied, n = 15 in Bodganvo’s study^18^ vs. n = 6 in our study, might have had an impact on the power of the analysis. Nevertheless, our data support the idea that retinal blood flow dysregulation precedes the development of neural dysfunction during the progression of type 2 diabetes. Our findings also indicate that the retina exhibits an intrinsic ability to maintain a steady resting blood flow but fails to react to metabolic disturbances by adjusting blood flow accordingly during diabetic insult.

The current study has some limitations. First, the study was performed under anesthesia with isoflurane but the impact of this anesthetic on retinal blood flow regulation is unclear. Because isoflurane, like other general anesthetics, can suppress central nervous activity and cardiovascular system, its broad impact on circulation is expected under high concentrations. Unfortunately, to the best of our knowledge, there was no study to examine the influence of isoflurane on retinal blood flow regulation. However, a previous study reported that there is no substantial difference in mouse ERG parameters between isoflurane and ketamine anesthesia^62^. In the present study, it is worth noting that all data were derived and compared under experimental conditions with the same level of anesthesia, which did not alter systemic or ocular parameters across different ages (Figs. 1 and 2). Therefore, we believe that isoflurane anesthesia might only have had a little effect, if any at all, in our study. Second, it is recognized that db/db mice are one of the most widely used animal models for type 2 diabetes research by developing hyperglycemia, hyperphagia, obesity, hyperinsulinemia, insulin resistance, and hyperlipidemia. However, it should be cautious to extend and translate our current findings to human application because there are some dissimilarities in phenotypes and pathophysiology between db/db mice and human patients with advanced diabetes^63^.

In conclusions, we found that the retina fails to regulate blood flow in response to systemic hyperoxia and flicker stimulations at the early stage of type 2 diabetes in db/db mice without apparent changes in resting retinal perfusion. Although the resting blood flow is maintained, retinal blood flow dysregulation in response to metabolic disturbances is manifested before a noticeable change in neuroretinal function. Although the underlying mechanisms responsible for retinal neurovascular uncoupling in diabetes remain largely unexplored, early detection and treatment of this flow dysregulation might help to preserve retinal tissue from developing irreversible retinopathy.

## Materials and methods

### Animal preparation

The Nihon University Ethical Committee approved the animal experiments, which were carried out according to the tenets of the Association of Research in Vision and Ophthalmology. We also confirmed that this study was performed in accordance with ARRIVE guidelines (https://arriveguidelines.org).

The 7-week-old male C57BL/KsJ-db/db mice (BKS.Cg-Dock7^m^+/+Lepr^db^/J; n = 6) and db/m (congenic nondiabetic littermates, n = 6) control mice were acquired from Charles River Laboratories JAPAN, Inc. (Yokohama, Japan) one week before the study. We used only male db/db mice because diabetes is more severe in male than in female db/db mice^63^. Blood glucose levels were measured from the tail vein (glucose assay kit; Abbott Laboratories, Abbott Park, IL). Mice were housed in a temperature-controlled room with a 12-h dark and light cycle with free access to food and water. Throughout the experiment, the mice were anesthetized with continuous inhaled 2% isoflurane (Pfizer, Tokyo, Japan) at a flow rate of 1.5 L/min. The pupils were dilated with 0.5% tropicamide (Santen Pharmaceutical Co., Osaka, Japan). Rectal temperature was measured and a heated blanket was used to maintain temperature between 37 °C and 38 °C.

### Systemic blood pressure and intraocular pressure measurements

Systemic blood pressure (BP) and intraocular pressure (IOP) were measured at 30 min following anesthesia induction. An automatic sphygmomanometer (THC-31, Softron, Tokyo, Japan) was used to measure systolic BP (SBP) and diastolic BP (DBP) at the tail. The IOP was measured by a handheld tonometer (TonolabTV02, ME Technical, Tokyo, Japan). The mean arterial BP (MABP) was derived from the standard formula: MABP = DBP + (SBP-DBP)/3. Ocular perfusion pressure (OPP) was calculated using the formula OPP = MABP − IOP^64^, due to the animals' prone position during the experiments.

### Retinal blood flow measurement

Retinal blood flow was measured with the LSFG-Micro system (Softcare Co., Ltd., Fukutsu, Japan), which is designed for small animals^64^. The LSFG-Micro system is equipped with a standard charge-coupled device camera (700 × 480 pixels) and a diode laser (830-nm wavelength) attached to a stereomicroscope (SZ61TR, Olympus Corporation, Tokyo, Japan). The underlying principle of the LSFG-Micro is the same as that of LSFG, which has been used in humans^65^ and animals^64^ for quantitative estimation of ocular (optic nerve head, choroid, and retina) blood flow. In brief, the mean blur rate (MBR) represents a relative index of blood velocity and is generated from the blurring of the speckle pattern formed by the backscattered light of the coherent laser by moving blood cells. The MBRs acquired from the vascular area around and at the optical nerve head (ONH) (Fig. 1B) reflect the entire retinal circulation and are used as an index of retinal blood flow^66^.

In the present study, the MBRs were obtained as follows: The mice were positioned on a stand with the right eye facing downward*.* A cover glass was gently placed on the left cornea with a drop of viscoelastic material. The margin of the ONH was indicated by manually placing a rubber o-ring (1.37-mm diameter) over the ONH fundus image. The MBRs were acquired within the o-ring area continuously at 30 frames/second. The vessels' average MBR was analyzed with an LSFG analyzer software (version 3.2.19.0, Softcare Co., Ltd., Fukutsu, Japan).

### Induction of systemic hyperoxia

Systemic hyperoxia was induced by inhalation of 100% oxygen over 10-min, as described in our previous studies^11,64^. The baseline value was determined as the mean of three consecutive flow measurements, obtained at 1-min intervals for 3 min before the initiation of hyperoxia. Retinal blood flow measurements were made every minute for 20 min during hyperoxia (10-min stimulation) and after the termination of hyperoxia (10-min recovery)^64^.

### Flicker light stimulation

We used a 12 Hz-flicker stimulation as this frequency triggers a maximal response of retinal blood flow in mice^64^. The ambient light was reduced to 1 lx or less before induction of flicker stimulation. The mice were dark-adapted for 2 h, with a light intensity for flicker stimulation of 30 lx for the rod-dominant mouse retina, as reported previously^64^. The retinal blood flow was measured with 20 s intervals throughout both the 3-min flicker stimulation and the 3-min recovery. The baseline value was calculated using the mean of three consecutive flow measurements obtained in 1 min (20-s intervals) before initiation of flicker light stimulation.

### ERG recording

Prior to the ERG, the mice were dark-adapted for a minimum of 12 h and then transferred to a room with dim red light. The full-field ERGs were recorded with PuREC (Mayo, Inazawa, Japan) under systemic anesthesia with isoflurane. The ground electrode was attached at the tail, the reference electrode in the mouth, and bilateral corneal electrodes were placed on the corneal surface. The 3.0 cd s/m^2^ flash was used to achieve a maximal response of both the cones and rods as previously reported^64^. The amplitude of the a-wave was measured from the baseline of the a-wave to the most negative trough, and the amplitude of the b-wave was measured from the trough of the a-wave to the positive peak of the b-wave^64^. The implicit times of the a- and b-wave were measured from the onset of the stimulus to the trough of the a-wave and from the trough of the a-wave to the peak of the b-wave, respectively. Oscillatory potentials (OPs) are small high-frequency oscillation wavelets superimposed on the b-wave’s ascending limb. The OP wavelets were labeled as OP1 to OP3 consecutively, starting at the first detected positive peak. The amplitudes (peak positive amplitude – peak negative amplitude of previous peak) and implicit times of OPs were measured^67^. The OP amplitudes were calculated by adding the first 3 positive wavelets and presented as ΣOP amplitude^9,18,68^.

### Experimental protocols

We performed the following protocols in each animal for longitudinal assessments of retinal blood flow regulation and neural function on three consecutive days, every 2 weeks from 8 to 20 weeks of age. The systemic hyperoxia response of retinal blood flow carried out on day 1, and the following day, the response to flicker light stimulation was conducted. The ERG recording was made on day 3. We verified that the systemic BP, IOP, and OPP were not altered by hyperoxia or flicker light stimulation in mice in a previous study^64^. An independent masked observer (AK) performed all data calculations and analyses.

### Statistical analysis

Data are expressed as mean ± standard error of the mean, and n value represents the number of animals studied. Retinal blood flow changes were calculated as percentage changes from the baseline. Normality of data distribution was verified by the Kolmogorov–Smirnov test. The significance of experimental intervention across different time points within and between groups were analyzed by one-way or two-way repeated-measures analysis of variance (ANOVA). This was followed by the Dunnett’s test or Holm-Sidak test, where appropriate. A *P*-value < 0.05 was considered statistically significant.
